# Protein-Carbohydrate Interactions as Part of Plant Defense and Animal Immunity

**DOI:** 10.3390/molecules20059029

**Published:** 2015-05-19

**Authors:** Kristof De Schutter, Els J. M. Van Damme

**Affiliations:** Lab Biochemistry and Glycobiology, Department of Molecular Biotechnology, Ghent University, Coupure links 653, B-9000 Ghent, Belgium; E-Mail: Kristof.DeSchutter@UGent.be

**Keywords:** lectin, carbohydrate, plant defense, animal immunity

## Abstract

The immune system consists of a complex network of cells and molecules that interact with each other to initiate the host defense system. Many of these interactions involve specific carbohydrate structures and proteins that specifically recognize and bind them, in particular lectins. It is well established that lectin-carbohydrate interactions play a major role in the immune system, in that they mediate and regulate several interactions that are part of the immune response. Despite obvious differences between the immune system in animals and plants, there are also striking similarities. In both cases, lectins can play a role as pattern recognition receptors, recognizing the pathogens and initiating the stress response. Although plants do not possess an adaptive immune system, they are able to imprint a stress memory, a mechanism in which lectins can be involved. This review will focus on the role of lectins in the immune system of animals and plants.

## 1. Introduction

Protein glycosylation is generally recognized as one of the major co- and post-translational modifications. Furthermore, many proteins that specifically recognize and bind these glycosylated structures have been identified in all kingdoms of life. In recent years, it has been clearly established that interactions between carbohydrate chains and their partner proteins can mediate many important biological events. These carbohydrate-binding proteins will not only interact with the glycan structures on proteins, but will also recognize and bind to carbohydrate chains on glycolipids and proteoglycans, or can interact with polysaccharides and free sugars.

Proteins, of non-immune origin, that can recognize and bind specific carbohydrate structures are classified as lectins. The term lectin (from the Latin ‘*lectus*’—chosen, selected) was first used by Boyd and Shapleigh [1] to emphasize the selectivity in the carbohydrate binding. More recently, Peumans and Van Damme [2] defined lectins as “all proteins possessing at least one non-catalytic domain which binds reversibly to a specific mono- or oligosaccharide”. The importance of these carbohydrate-binding proteins is shown by their occurrence in all kingdoms of life.

The whole group of lectins can be divided into multiple families of structurally and evolutionary related proteins based on a conserved carbohydrate-recognition domain (CRD). In plants, lectins can be classified into 12 families [3] (Table 1). This classification system proved to be superior to the many attempts to group plant lectins based on sugar specificity and allows to classify the majority of all putative lectin sequences based on the sequence and evolutionary relationships between the CRDs. The assignment of animal lectins to several groups is less straightforward. Originally animal lectins were divided into the C-type (Ca^2+^ dependent) lectins and the S-type (sulfydryl-dependent or B-galactoside binding) lectins [4], and all remaining lectins were classified in a heterogeneous group, the N-type glycans (not C nor S-type). However, as more knowledge became available with respect to the three-dimensional structure, lectin activity was found to be associated with a diversity of structural motifs. At present, there are at least 14 families grouping structurally related carbohydrate-binding domains (Table 1) and many other “orphan” lectins with a unique structure. Some of these orphan lectins belong to well-established families that are generally not linked to sugar-binding activity [5]. Furthermore, many animal lectins will also interact with macro-molecules through protein-protein, protein-lipid or protein-nucleic acid interactions [6]. Of all lectin domains distinguished in animals only the families with calnexin and calreticulin and the R-type lectins are also present in plants.

In addition to the classification based on structural domains, lectins can be grouped based on their expression patterns. In plants, lectins with a constitutive expression are often present at high concentrations in some specific cells or organs. Examples of these abundant lectins are the storage proteins found in seeds or specialized vegetative tissues. These lectins accumulate during a certain developmental stage and offer a source of nitrogen or amino acids that can readily be degraded when needed [3]. In addition to a role as storage lectins, the localization of these abundant proteins in the vacuoles or extracellular space, suggests a role in plant defense. Most probably, these lectins serve a role in protecting the seeds from pathogens and diseases, or herbivory.

In contrast to the highly abundant constitutively expressed lectins, the inducible plant lectins are present at a low basal level, often too low for detection by western blot analysis. However, in response to biotic or abiotic stresses, the expression of these lectins is upregulated. Although lectin levels rise significantly, they remain low abundant proteins. Interestingly, most of these stress inducible lectins are located in the nucleocytoplasmic compartment of the plant cell and are often expressed throughout the plant. Based on these observations, it is proposed that lectin-mediated protein–carbohydrate interactions in the cytoplasm and the nucleus play an important or possibly even crucial role in the stress physiology of the plant cell [7,8]. Similar to plants, several animal lectins are known to have an inducible expression pattern upon detection of stress [9]. Especially for the lectins with low expression levels, the study of protein-carbohydrate interactions can be challenging. Furthermore, the diversity of carbohydrates and glycosidic linkages complicates a quantitative study of carbohydrates using traditional methods [10]. However, novel computational methods can circumvent some of these difficulties and provide new tools to study carbohydrate interactions [10].

**Table 1:** Lectin families in plants and animals.

**Plant Lectin Family**	**Typical Saccharide Ligands**	**Predicted Localization**
*Agaricus bisporus* lectin family	GlcNAc/GalNAc, Galactose	Nucleus, cytosol
Amaranthin family	GalNAc	Nucleus, cytosol
Chitinase related agglutinin family	High mannose N-glycans	Vacuole, membrane bound
Cyanovirin family	Mannose	Nucleus
*Euonymus europaeus* lectin family	Galactosides, high-mannose N-glycans	Nucleus, cytosol
*Galanthus nivalis* lectin family	Mannose	Vacuole, nucleus, cytosol or membrane bound
Hevein family	Chitin	Vacuole
Jacalin family	Mannose- and galactose-specific subgroup	Nucleus, cytosol, vacuole
Legume family	Mannose	Vacuole, nucleus, cytosol or membrane bound
LysM family	Chitin, peptidoglycan	Vacuole, nucleus, cytosol or membrane bound
*Nicotiana tabacum* lectin family	(GlcNAc)_n_, high-mannose and complex N-glycans	Nucleus, cytosol
Ricin-B family	Gal/GalNAc, Sialylated Gal/GalNAc	Vacuole, nucleus, cytosol
Animal Lectin Family *	**Typical Saccharide Ligands**	**Predicted Localization**
Calnexin and Calreticulin	Glc_1_Man_9_	ER
M-type lectins	Man_8_	ER
L-type lectins	Various	ER, Golgi
P-type lectins	Man_6_-phosphate	Secretory pathway
C-type lectins	Mannosides, galactosides, sialic acids and others	Membrane bound, extracellular
S-type lectins (galectins)	β-galactosides	Cytosol, extracellular
I-type lectins (siglecs)	Sialic acid	Membrane bound
R-type lectins	Various	Golgi, membrane bound
F-box lectins	GlcNAc_2_ of N-glycans	Cytoplasma
Fibrinogen-type lectin	GlcNAc, GalNAc	Membrane bound, extracellular
Chi-lectins	Chito-oligosaccharides	Extracellular
F-type lectins	Fucose terminating oligosaccharides	Extracellular
Intelectins	Galactose, galactofuranose, pentoses	Membrane bound, extracellular
Annexins	Glycosaminoglycans, heparin and heparin sulfate	Membrane bound

* Table redrafted from [6,11].

The binding of a lectin with a specific carbohydrate structure is mediated through hydrophobic (Van der Waals) interactions and hydrogen bonds. The specificity of this interaction results from the formation of specific hydrogen bonds and metal coordination bonds to key hydroxyl groups [12]. In addition, unwanted recognition is sometimes excluded by steric exclusion. However, the sequence or the threedimensional conformation of the CRD is no indication for its specificity, since structurally unrelated lectins can recognize similar carbohydrate structures [12]. In addition some lectins with similar CRDs can recognize different carbohydrates. These phenomena can be attributed to the shallowness of the sugar-binding site and the limited number of contacts with the sugar that allows the CRD to recognize multiple carbohydrate structures, further referred to as ‘the promiscuity of the CRD’. Another interesting feature in carbohydrate-binding sites is that within a lectin family, there is a common mechanism for binding of a core monosaccharide in the primary binding site, but diversity in binding of oligosaccharides or glycoconjugates is achieved through extended and secondary binding sites unique to individual lectins [13]. Most likely, a number of lectin domains from animals and plants descended from a common ancestor through divergent evolution [13]. One lectin family that is represented both in mammalia and plants is the R-type lectin family (Ricin-B family in plants). The β-trefoil structure of the R-type lectin was first identified in ricin. In animals a structurally related domain was found in fibroblast growth factors and the cysteine-rich domain of the mannose receptor [14]. Although both ricin and the mannose receptor can bind glycan structures containing galactose ((sialylated) galactose/*N*-acetylgalactosamine and Lewis^a/x^ structures respectively), their carbohydrate binding site differs significantly [14]. While the β-trefoil structure is conserved, the sequence of their CRDs differs, and amino acids involved in the binding of the carbohydrate structure are not conserved, accounting for the promiscuity of the CRDs.

Animal and plant lectins tend to play a role in a wide variety of biological processes, with some lectins having more than one function. Unfortunately for many lectins their physiological importance remains enigmatic. Several CRDs are involved in the recognition of invaders, and thus are part of the immune system, which consists of various types of cells and molecules that specifically interact with each other to initiate the host defense mechanism. To operate properly, the immune system must be able to detect a wide variety of pathogenic agents, and distinguish them from the organisms own healthy cells. Apart from their role in the immune system CRDs are important for a multitude of cellular processes like cell-cell interactions, self/non-self-recognition and intracellular routing. In addition, lectins also play an important role as molecular chaperones for glycoprotein quality control.

This review wants to offer an anthology of the multitude of functions carried out by lectins in the immune system of plants and animals. We will discuss the role of lectins in the recognition of pathogens in the innate immune system as pattern recognition receptors, their involvement in autophagy and their importance in stress signaling and other processes in the immune system. Furthermore we will also touch on their contribution in the vertebrate adaptive immune response and their potential involvement in epigenetic stress imprinting in plants. However, in view of the vast amount of lectins known today this review will only focus on a few examples to illustrate the functions of lectins in the immune system, and does not claim to present a complete overview of all the lectins involved.

## 2. Lectins as Pattern Recognition Receptors in the Innate Immune System

The first line of defense against infection by other organisms is the innate immune system. This non-specific immune system consists of cells and molecules that can recognize the pathogens and initiate a generic defense response. This system does not offer long-lasting protective immunity, as does the adaptive immune system, but activation of the innate system is required to initiate an adaptive response. In the innate immune system, pathogenic microorganisms are recognized through highly conserved structures, pathogen associated molecular patterns (PAMPs). These structures are recognized by the pattern recognition receptors (PRRs) of the host [15]. Since many of the PAMPs recognized by the PRRs are carbohydrate structures, lectins play an important role as PRRs. These lectin PRRs are highly variable in structure and can occur in a soluble as well as in a membrane-associated form.

### 2.1. Animal Lectin PRRs

**Figure 1:**
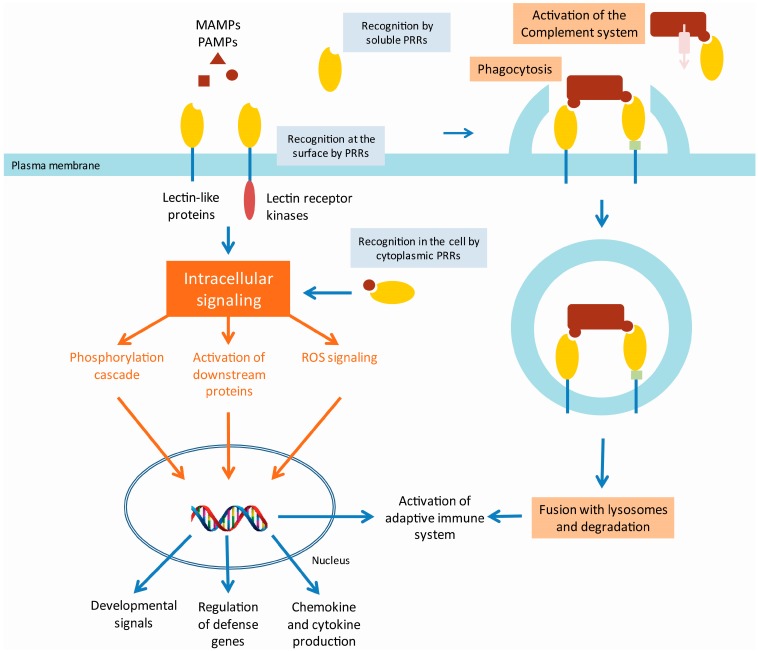
Model of animal innate immunity by lectin PRRs. Detection of pathogen/microbe-associated molecular patterns (P/MAMPs) by membrane bound pattern recognition receptors (PRRs) with lectin domains (lectin receptor kinases and lectin-like proteins) will initiate an intracellular signaling cascade on the one hand and lead to phagocytosis of the pathogen on the other hand. Soluble lectin PRRs can recognize P/MAMPs and subsequently bind to receptors that will trigger phagocytosis or can activate the complement system. Next to the recognition of P/MAMPs at the surface, cytoplasmic PRRs can sense the presence of bacterial peptidoglycan fragments which leads to activation of the intracellular signaling cascade. The intracellular signaling cascade can include downstream protein phosphorylation (e.g., MAPK cascade, TRAF/IRAK signaling), transcription factor activation (e.g., NF-kB, AP1, NFAT), or reactive oxygen signaling with cross-communication between the different components. This signaling cascade will lead to activation of stress-responsive or developmental signal-responsive genes or the production of chemokines and cytokines. In addition, the innate immune response can activate the adaptive immune response.

One of the best studied examples of an inducible lectin with a role as a PRR in the immune response is the mannose-binding protein (MBP), also known as mannose binding lectin (MBL) 2. This lectin is a family member of the collectins which represent a group of soluble lectins belonging to the Ca^2+^ dependent C-type lectin superfamily [16]. Collectins are organized as oligomers of a trimeric subunit, composed of a C-terminal lectin domain and an N-terminal collagen-like domain which enables the formation of the triplets [16]. Under normal conditions, MBL2 is synthesized at a basal level by the liver and secreted into the serum. However, after exposure to pathogenic microorganisms, the level of MBL2 mRNA transcripts and protein increases [17]. In response to infection, local inflammatory cells secrete cytokines into the bloodstream that stimulate the liver to produce large numbers of acute phase proteins, including MBL2. In the innate immune system, MBL2 functions as a PRR binding a range of carbohydrates including *N*-acetylglucosamine, mannose, *N*-acetylmannosamine, fucose and glucose, enabling the lectin to interact with surface glycans on a wide selection of viruses, bacteria, yeasts, fungi and protozoa (Figure 1).

When the C-terminal recognition portion of MBL2 binds to carbohydrates on the pathogen surface, the N-terminal domain can interact with collectin receptors on macrophages, which in turn leads to phagocytosis (Figure 1). In addition, MBL2 can activate the complement pathway through a unique pathway, *i.e.*, the lectin pathway, independent from the classical pathway [18] (Figure 1). In the classical pathway of complement fixation, the binding of antibodies to pathogens leads to the recruitment of the first component of complement, C1q. In turn, C1q associates with two serine proteases, C1r and C1s, which initiates a proteolytic cascade. In the lectin pathway, MBL2 activates the same cascade through direct activation of serine proteases, MBP-associated serine proteases (MASPs), without the involvement of C1q. In essence, MBL2 is substituting for C1q [19]. The structural organization of collectins resembles that of C1q, being composed of an N-terminal collagenous domain and a globular C-terminal domain. However, C1q differs from MBL2 in that it contains a C-terminal immunoglobulin binding domain. However, at present MBL2 is the only collectin known to activate the complement pathway [16]. Activation of C2-C4 complexes through activation of the proteolytic cascade from either pathway leads to the cleavage of the C3 complement component. The resulting C3b fragment will insert into the surface of the target, initiating a lytic pathway in which additional complement components insert into the membrane to form a pore. In addition, C3b mediates phagocytosis of the pathogen through interaction with receptors on macrophages.

The pentraxins (belonging to the L-type lectin superfamily) represent another class of soluble lectins involved in the innate immune response, among these are C-reactive proteins (CRP) and serum amyloid P components (SAP) [20,21]. Similar to MBL2, they are acute phase proteins whose expression levels are increased upon stimulation by cytokines (IL-1 and IL-6). Their biological functions include activation of the complement pathway and stimulation of phagocytic leukocytes [16]. Furthermore ficolins are complement activating soluble PRR able to sense molecular patterns on both pathogens and apoptotic cell surfaces [6]. 

The mannose receptor is a membrane bound PRR on the surface of macrophages [22]. This glycoprotein belongs to the C-type superfamily of lectins and possesses eight CRDs [23,24], which bind terminal mannose, fucose and *N*-acetylglucosamine (GlcNAc) residues [25]. Binding of the mannose receptor to components in the cell wall of pathogens leads to the internalization of the pathogen by macrophages (Figure 1). After phagocytosis by the macrophage, the phagosomes will fuse with lysosomes, killing the pathogen. However, Mycobacteria, the causal agent of tuberculosis and leprosy, make use of the mannose receptor to gain access into the cell. They prevent the fusion of the phagosome with lysosomes, creating a habitat to survive and proliferate within macrophages [26].

In addition to its role as a PRR in the innate immune response, several other functions have been attributed to this mannose receptor, including the attachment of sperm to oocytes [27], endocytosis for antigen presentation [22] and clearance of glycoproteins [28,29]. Interestingly, the expression of the mannose receptor is repressed during the early stages of inflammation and is induced during the resolution phase [30]. This expression profile is in accordance with a role in clearing inflammatory agents [30].

Recognition of yeasts through binding of the β-glucans on their surface by Dectin-1, a natural killer (NK)-cell-receptor-like C-type lectin, leads to the uptake and killing of these yeasts. In addition, Dectin-1 will induce a signaling cascade leading to the production of cytokines and chemokines (Figure 1). This signaling occurs cooperatively with Toll-like receptors (TLR) and the use of spleen tyrosine kinases (SYK) [31,32].

The nucleotide-binding oligomerization domain (NOD) proteins NOD1 and NOD2 provide the cell with another level of microbial surveillance. Being cytoplasmic PRRs they recognize conserved fragments in particular muramyl peptides in the cell wall of many types of bacteria [33]. These muramyl peptides are derived from the bacterial peptidoglycan. The mechanisms by which the muramyl fragments enter the cell and activate the NOD-like receptors NOD1 and NOD2 remain poorly understood but multiple routes of entry have been reported [33]. Upon sensing distinct peptidoglycan fragments, NOD1 and NOD2 activate intracellular signaling pathways that drive pro-inflammatory and antimicrobial responses [33]. In addition, polymorphisms in NOD2 have been identified as the strongest genetic risk factor in the development of Crohn’s disease [33]. 

### 2.2. Plant Lectin PRRs 

**Figure 2:**
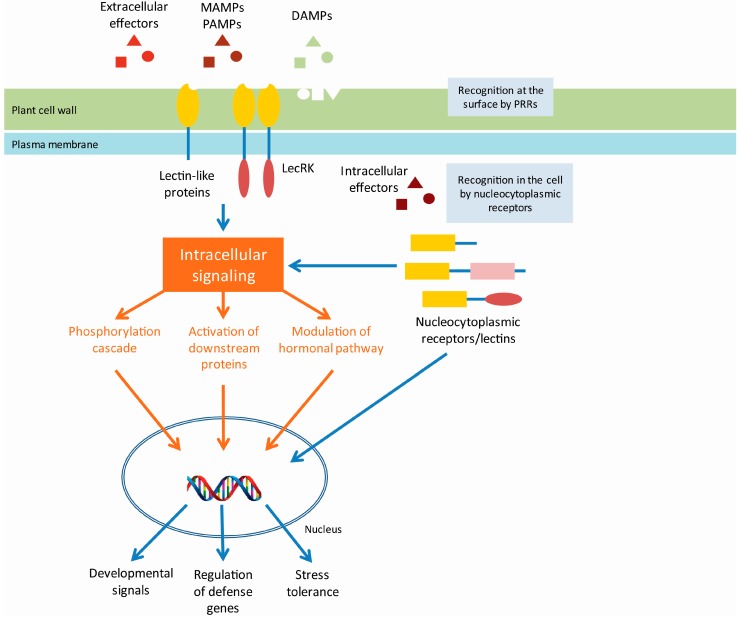
Model of plant innate immunity by lectin PRRs. After perceiving extracellular signals (pathogen/microbe-associated molecular patterns (P/MAMPs), damage associated molecular patterns (DAMPs) or pathogen-derived effector proteins) by membrane bound pattern recognition receptors (PRRs) with lectin domains (lectin receptor kinases LecRKs and lectin-like proteins), an intracellular signaling cascade is initiated in plants. This signaling cascade can include downstream protein phosphorylation, transcription factor activation, or modulation of hormonal pathways, ultimately leading to activation of stress-responsive or developmental signal-responsive genes and pathogen/microbe triggered immunity (P/MTI). In addition, perception of intracellular effectors by nucleocytoplasmic receptors (potentially including nucleocytoplasmic lectins), leads to activation of the effector triggered immunity (ETI). However, the precise signaling pathways leading to P/MTI and ETI are not fully known and need to be elucidated. Figure redrafted from [40,42].

Despite obvious differences in the immune system of plants and animals, there are also some striking similarities [34]. While the adaptive immunity is unique to vertebrates, the innate immune response most probably has ancient origins [35]. In contrast to animals where specialized cell types (macrophages, neutrophils and dendritic cells) in the blood circulatory system play a key role in the detection of pathogens and the activation of the immune system, most plant cells are autonomously capable of sensing the presence of pathogens and activating a defense response [36]. Similar to animals, plants have evolved systems for non-self-recognition and anti-microbial defense [36]. Like animals, plants have acquired specialized PRRs for their defense against pathogens [37]. These PRRs are able to recognize the damage-associated molecular patterns (DAMPs) and the pathogen- or microbe-associated molecular patterns (PAMPs/MAMPs). As in animals, many of these PRRs carry lectin domains able to recognize and interact with carbohydrate structures from microbial organisms or saccharides derived from plant cell wall damage. Upon PAMP/MAMP and DAMP perception by the PRRs, an intracellular response is activated, referred to as the PAMP/MAMP-triggered immunity (PTI/MTI) (Figure 2). Besides the components to sense the pathogens, also the building blocks of PAMP-induced signaling cascades leading to transcriptional activation of response genes are shared between the two kingdoms. In particular, nitric oxide as well as mitogen-activated protein kinase (MAPK) cascades have been implicated in triggering innate immune responses, which ultimately lead to the production of anti-microbial compounds [36]. In addition, this response includes ion fluxes across the plasma membrane, increase of cytosolic Ca^2+^ levels, production of reactive oxygen species and protein phosphorylation. This complex response of the plant finally leads to profound transcriptional changes, stomatal closure and cell wall reinforcement [38], and will ultimately limit the growth of the pathogen [39]. 

The best studied example of the perception of PAMPs by plant PRRs is the recognition of bacterial flagellin, through the conserved *flg22* epitope, by the Arabidopsis FLS2 receptor-like kinase [38]. For more detailed information on FLS2 signaling and some other examples involving recognition through protein-protein interactions we refer to some recent review papers focused on this topic [40,41]. A significant part of the patterns recognized by the plant PRRs are carbohydrate structures, which are either present on the surface of the pathogen (e.g., lipopolysaccharides, peptidoglycans and chitin molecules) or are derived from plant own molecules (*i.e.*, DAMPs), released during invasion of the pathogen (e.g., cellulose fragments, arabinogalactan proteins and oligogalacturonides).

For the recognition of the D/M/PAMPs, a large diversity of membrane-bound or soluble PRRs with a lectin domain have been identified in plants. However, only a limited number of them have been functionally characterized. The membrane-bound PRRs carrying one or more extracellular lectin domains are often coupled to an intracellular Ser/Thr kinase domain (Figure 2). These lectin receptor kinases (LecRK) can be classified into four types: G-, C-, L- and LysM-type [42,43].

Due to their sessile lifestyle, plants are not only subjected to biotic stresses but also to multitude of abiotic stress factors (e.g., cold, excess water, increased salt concentrations). To be able to respond to these detrimental environmental conditions, plants have evolved defense pathways able to sense and respond to these abiotic stresses. Several of the LecRKs have been reported to act both upon biotic and abiotic stresses [44,45,46,47,48,49].

In contrast to animal systems, where the C-type lectins represent a major player in the recognition of pathogens and the induction of the immune response, C-type lectins in plants are rather rare. Only one C-type LecRK has been identified in *Arabidopsis* and rice, and its function is not fully elucidated [45,50,51].

The G-type LecRKs carry a lectin domain belonging to the *Galanthus nivalis* agglutinin (GNA) family of plant lectins. Although 32 G-type LecRKs have been identified in *Arabidopsis* and 100 in rice [51], it still needs to be confirmed whether the lectin domain plays a role in the interaction with the pathogen. G-type LecRKs have been shown to function in self-incompatibility reactions and defense to both biotic and abiotic stresses [44,46,49].

The 42 L-type LecRKs identified in the genome of *Arabidopsis thaliana* (72 in rice) are grouped into nine clades on a dendrogram. These kinase receptors with a Legume-like (L-type) lectin domain show variable expression patterns in different tissues and developmental stages in response to stimuli [45]. The amino acid sequence of the legume-like lectin domain is only poorly conserved in the L-type LecRKs, therefore it is doubtful whether it possesses true lectin activity. Despite the fact that it remains to be proven that L-type LecRKs possess lectin activity, several of these proteins were reported to be involved in plant resistance to pathogens [47,52,53,54]. In addition, some L-type LecRKs have been described to act in hormone signaling and stomatal immunity [47]. Although the overall legume-like lectin domain is poorly conserved, a hydrophobic site within this lectin domain is highly preserved [53]. This site can play a role in the detection of extracellular ATP by L-type LecRKs. Recently, extracellular ATP was shown to play a role as a signaling molecule in plant stress responses [55,56].

The best studied LecRKs in plants belong to the LysM-type LecRKs. These receptors recognize the GlcNAc moieties in various types of bacterial peptidoglycans and fungal chitins [57,58]. Next to their role in recognizing pathogenic microorganisms, the LysM LecRKs are also involved in the recognition of beneficial microorganisms such as mycorrhiza and rhizobacteria [58,59,60].

### 2.3. Plant Effector Triggered Immunity 

In addition to the PTI/MTI responses, plants have evolved another defense mechanism, referred to as the effector triggered immunity (ETI) (Figure 2). In contrast with the former responses, ETI will mainly act inside the plant cell. Successful pathogens have evolved mechanisms to counter the PTI response of the plant by the delivery of specific elicitors or effectors, also called avirulence (Avr) proteins [61]. These effectors are usually directly secreted into the plant cell by a type III secretion system and suppress or block the PRR-dependent signaling [62]. These effectors are recognized by a class of intracellular plant receptors containing a nucleotide-binding and leucine-rich repeat domain (NB-LRR), thereby activating the ETI response. In the ETI response, plants express specific resistance genes upon recognition of these pathogenic effectors to overcome the action of these effectors. Both the PTI/MTI and the ETI response can lead to programmed cell death of the host cell through activation of the hypersensitive response (HR), but can also result in systemic acquired resistance (SAR) which activates defense mechanisms throughout the plant [38,63].

## 3. Autophagy 

Recognition of pathogens by PRRs leads to the induction of the immune responses and will also result in phagocytosis of the pathogen. These phagocytic vesicles will subsequently fuse with lysosomes leading to the degradation of the pathogen. However, some bacteria are capable of escaping this process by releasing effectors into the cytosol. *Mycobacterium tuberculosis* secretes an effector (EsxH) that prevents the maturation of the phagosome and fusion with lysosomes, thereby escaping degradation and creating a niche to proliferate [64]. In the case of the bacterium *Salmonella enterica* serovar Thyphimurium, the major cause of food poisoning, the pathogen can invade and grow in the gut epithelial cells after digestion by its host. *S*. *thyphimurium* is recognized by the Toll-like receptor 4 on the gut epithelial cells, which binds to the lipopolysaccharides on the outer membrane of the Gram-negative bacterium [65]. *Salmonella* uses this interaction and its subsequent phagocytosis to invade the host cell. Early after cell invasion, the bacterium resides in a vesicle known as the *Salmonella* containing vacuoles (SCV). The bacteria then use a type III secretion system to generate pores in the SCV membrane, through which they can deliver effectors into the cell’s cytoplasm [66]. These effectors modulate the activity of the host cell machinery to prevent degradation and promote growth of the intracellular pathogen. Moreover, some bacteria can escape the damaged SCVs and replicate in the cytosol. However, cells have developed a defense mechanism against this kind of intracellular pathogens. Microbes in damaged vesicles as well as microbes residing in the cytosol are targeted by the autophagy system that will engulf them in autophagosomes and restrict bacterial growth [67,68]. Autophagy is a strictly regulated and mainly non-selective process used by the cell to capture cytoplasmic cargoes in a double membrane vesicle, the autophagosome, to maintain a balance between synthesis and degradation of their own components. The uptake of the intracellular cargo in these vesicles is mediated through evagination of the rough endoplasmatic reticulum associated membrane [69]. When this vesicle fuses with lysosomes its contents become degraded and nutrients and building blocks get recycled. Malfunctioning of the autophagy system has been linked to human diseases such as cancer, neurodegenerative disorders, diabetes and inflammatory bowel disease [66]. In addition to its function in the housekeeping of the cell, autophagy can also help to defend the mammalian cytosol against bacterial infection [70]. Efficient pathogen engulfment is mediated by cargo-selecting autophagy adaptors that rely on pattern recognition or danger receptors to label the invading pathogen as autophagy cargo [71]. This labeling is typically mediated through polyubiquitin coating [72]. Recently the carbohydrate-dependent galectin-8 pathway was discovered, representing a novel mechanism that allows to get rid of bacteria that have been taken up by the cell [71]. Galectins are β-galactoside binding lectins that accumulate in the cytosol before being secreted via a leader peptide-independent pathway [71]. When these galectins occur extracellularly, they bind to cell surface glycans in order to modulate cellular behavior. Inside the cytosol, where complex carbohydrates are absent under normal physiological conditions, galectins play a role as danger and/or pattern recognition receptors. Thurston *et al.* [71] reported that galectin-8 is recruited to damaged SCVs and the absence of galectin-8 leads to an increase in the growth rate of the intracellular bacteria. However, in contrast to the direct tagging of *Shingella flexneri* by autophagy related gene 5 (ATG5) [73], it was observed that galectin-8 does not bind to the bacterial glycans but to the host cell’s own complex glycans that become accessible in the damaged vesicles. Supporting this observation, galectin-8 was also found to decorate vesicles with other vesicle damaging bacteria such as *Listeria monocytogenes* and *Shigella flexneri*. Moreover, osmotic damage of cytoplasmic vesicles also leads to the recruitment of galectin-8 [71]. Galectin-8 interacts with the autophagy receptor NDP52, which subsequently recruits LC3 and other components of the autophagy machinery to the damaged vesicles. Although, other galectins (galectin-3 and -9) are also recruited to the damaged vesicles, their role in cellular defense to infection remains enigmatic [71].

Similar to animals and yeast, essential autophagic machineries are conserved in plants. The role of autophagy related genes (ATGs) in the lifecycle of plants is similar to that in animals. In *Arabidopsis*, autophagy is activated in certain stages of development, senescence or in response to starvation, or after environmental stress [74]. In addition, autophagy in plants is suggested to be required for proper regulation of the hypersensitive response programmed cell death [75,76]. However the question remains whether autophagy is involved in the elimination of bacterial pathogens in plants and if any of the cytoplasmic lectins contribute to this process. Compared to animals, not many examples of intracellular microorganisms are known in plant cells since due to their rigid cell wall, plant cells are usually not phagocytic [77]. One well studied example in plants is the endosymbiosis of *Rhizobium*, where the engulfment of the microorganism by a plant-derived membrane occurs by a phagocytic mechanism [78]. These endosymbionts counteract their degradation [79] but it is unknown if they trigger an autophagosomic response. One indication that cytoplasmic plant lectins can be involved in autophagy comes from the observation of an interaction between the *Arabidopsis* jacalin-related lectins and proteins of the ER-bodies [80]. These novel ER-derived organelles have been shown to play a role in plant defense against pathogens [81] and are linked to autophagocytosis [82].

Next to a role in the defense against intracellular bacteria, autophagy has also been reported to play a role in the elimination of intracellular and extracellular viruses. Overexpression of *beclin 1*, a protein required for autophagosome formation and recruitment of autophagosome components*,* in neurons reduced Sindbis virus replication [83], while in cells deprived of *beclin 1*, Sindbis virus and *Herpes simplex* virus replicate to higher titers than in wild-type cells [84,85]. Similarly, the plant tobacco mosaic virus accumulated to a higher level in *Beclin 1*-silenced plants than in wild-type plants [75]. These results indicate that autophagy functions as an antiviral defense mechanism in which the ATG proteins might target cellular factors or pathways required for virus replication and spread [86].

## 4. Non-PRR Function of Lectins in the Immune Response

Many of the animal PRRs that detect microbial infection induce the innate immune response by triggering an intracellular signaling cascade which stimulates expression of chemokines, cytokines and other immune mediators. The role of protein-carbohydrate interactions is not limited to the interaction between the pathogen and the host cell, but they can also play an important part in the signaling pathway induced upon pathogen recognition or in other processes of the immune response. 

Inflammation is one of the responses of the innate immune system upon detection of stress. Induction of stress signaling in macrophages upon recognition of microorganisms leads to the secretion of chemokines and cytokines which initiate inflammation. A key function of inflammation is the recruitment of leukocytes through vascular tissue, a process in which C-type lectins play a key role. Selectins are present on the surface of leukocytes (L-selectins), endothelial cells (E- and P-selectins) and platelets (P-selectins) and bind to sialyl Lewis^x^ structures (can also bind other carbohydrates such as heparin) [87]. The interaction between these selectins and their ligand will result in a process called leukocyte rolling, which will slow down the movement of the leukocytes in the bloodstream at the sites of inflammation and increases the contact between the leukocyte and the epithelial cells. This allows the tight interaction between Integrin (MAC-1) and its ligand (IMAC-1). In addition, MAC-1 is also able to bind to carbohydrates such as zymosan and lipopolysaccharides [16].

Sialic acid binding Ig-like lectins or Siglecs are a good example of lectins involved in regulating cellular activation within the immune system. Sialoadhesin (siglec-1), a macrophage lectin-like adhesion molecule, has been implicated in key interactions with T-lymphocytes, leading to generation of cytotoxic T-cells [88]. Since siglec-1 lacks the cytoplasmic tyrosine-based motif present in other siglecs, it is suggested to mediate extracellular rather than intracellular functions [89]. The B-cell restricted Siglec-2 (CD22) plays an important role in regulating B-cell activity [89]. The balance between positive and negative signals is an important factor in the immune response [90]. Upon cellular activation by receptors, counteracting inhibitory signals can be delivered through distinct mechanisms, resulting in a higher activation threshold [89]. CD22 is described as an inhibitory receptor. Although the precise role of the sialic acid binding activity of CD22 remains to be elucidated, mice lacking CD22 show a higher prevalence of autoantibodies with increasing age [91].

Similar to animals, several families of plant lectins have been identified with a putative role in plant defense against biotic or abiotic stress other than the recognition of stress agents. Under non-stress conditions, the expression levels for these lectins are low, often under the detection limit. But after perception of biotic and/or abiotic stress signals, their transcript levels are upregulated. Although the precise role of these lectins in plant defense is not fully understood, several of them have been reported to increase plant resistance to biotic or abiotic agents. 

Lectins with an *Euonymus* lectin-like (EUL) domain are ubiquitous in plants, indicating a universal role for these lectins [92]. Despite their widespread distribution, the EUL proteins were only recently described as a new lectin family. Although all characterized lectins from this family show a very similar localization in the nucleocytoplasmic compartment of the cell, their ligand specificity varies depending on the protein and species studied [93]. Most EULs are chimeric proteins in which the lectin domain is linked to an unknown domain. In contrast to the *Euonymus europaeus* agglutinin, the prototype of this family, which is expressed at high concentrations in the arils surrounding the seeds, the EUL proteins from *Arabidopsis* and rice are low abundant proteins, the expression of which is induced after the plant was subjected to various biotic (bacterial and fungal infection) and abiotic (dehydration, salinity, osmotic stress and abscisic acid (ABA) treatment) stresses. *Arabidopsis* EULS3 was shown to interact with the nucleocytoplasmic ABA receptor RCAR1 [94] and a Ca^2+^ dependent kinase involved in ABA response in stomata guard cells [95], suggesting a role for ArathEULS3 in ABA signaling and stomatal closure.

Jacalin-related lectins can be divided into two subgroups based on their substrate specificity: the galactose-binding and the mannose-binding jacalins [3]. Structural analysis demonstrated that the relatively large size of the carbohydrate binding site, which is extended in the galactose-binding jacalins compared to the mannose-binding jacalins, allows this promiscuity of the CRD [96]. There are some striking differences between the two subgroups: First, polypeptides of the mannose-binding jacalins remain intact whereas those of the galactose-binding subgroup are cleaved into two dissimilar polypeptides [3]. Second, galactose-binding jacalins are synthesized on ER bound ribosomes as a proprotein whereas the mannose-binding lectins are synthesized without signal peptide [3], resulting in a different subcellular localization for both subgroups of jacalin-related lectins. Whereas the galactose-binding jacalins mainly reside in the vacuole, the mannose-binding lectins are located in the nucleocytoplasmic compartment of the cell [97]. Several jacalin-related lectins have been associated with disease resistance, abiotic stress signaling, wounding and insect damage [98]. The jacalin domain is often present as part of chimeric proteins, where the lectin domain is fused to an unrelated domain. Interestingly, several of these chimeric proteins contain domains related to stress response and defense. In Poaceae species (wheat, rice and maize), mannose-binding jacalin-related proteins in which the C-terminal lectin domain is fused to an N-terminal dirigent or disease-response domain have been identified. In wheat (*Triticum aestivum*), nearly half of the jacalin-related lectins are chimeric proteins containing this dirigent motif, and those already studied in more detail were all reported to have increased expression levels after application of some stress factor [98]. Rice (*Oryza sativa*) expresses a protein composed of a C-terminal jacalin domain fused to a leucine rich repeat (LRR) and an N-terminal NB-ARC domain. The NB-ARC domain is a novel signaling motif found in bacteria and eukaryotes, and is shared between plant resistance gene products and regulators of cell death in animals. Furthermore, also proteins consisting only of one or more jacalin domain(s) can be upregulated after stress perception. For example, Orysata is a salt inducible mannose-binding lectin from rice shoots. In addition, Orysata is also induced upon hormone treatment (jasmonic acid (JA) and ABA), infection with *Magnaporthe grisea* and during senescence [99,100,101]. The expression of the wheat TaJRLL1 lectin, a component of the defense signaling, is induced after fungal infection in a hormone (salicylic acid (SA) and JA) dependent manner [102]. In contrast to the plant hormone dependent regulation, jacalin-related lectins can also be regulated independently from hormones. The *Arabidopsis* JAX1 offers resistance to potex virus by inhibiting accumulation of viral RNA in a hormone independent manner [103]. In addition to a signaling role, jacalin-related lectins can have an effector function. The Helja lectin from sunflower (*Helianthus tuberosus*) was shown to possess antifungal properties. Furthermore, it induces morphological changes and reactive oxygen species production in yeast as well as alteration of membrane permeability [104].

Recently, proteins containing the jacalin-related lectin domain were also identified outside the plant kingdom. The human lectin ZG16p is a soluble mammalian lectin with a jacalin-related β-prism fold. ZG16p has been reported to bind both to glycosaminoglycans and α-mannose terminating short glycans [105]. Recognition of a broad spectrum of ligands by ZG16p may account for the multiple functions of this lectin in the formation of zymogen granules via glycosaminoglycan binding, and in the recognition of pathogens in the digestive system through α-mannose-related recognition [105]. Furthermore, the jacalin-related domain has also been reported in a few prokaryotes [3].

The expression of the nucleocytoplasmic tobacco lectin, referred to as the *Nicotiana tabacum* agglutinin or Nictaba, is enhanced after treatment with JA, a plant hormone important for plant development and involved in signaling of abiotic and biotic stress, such as insect herbivory [40]. Nictaba specifically recognizes GlcNAc oligomers but also reacts with the core structure of N-glycans. The Nictaba domain is widespread in the plant kingdom and several Nictaba-related lectins were shown to have a role in plant defense [40]. For instance, Nictaba was shown to possess entomotoxic activity on Lepidopteran insects and can protect plants against insect herbivory [40]. The phloem proteins (PP) represent a subgroup of the Nictaba-related lectins isolated from *Cucurbitaceae*. In contrast to Nictaba, the PP2 lectins are constitutively expressed in the companion cells of the phloem and are subsequently translocated to the phloem sap. Expression of the *Arabidopsis* homolog PP2-A1 is enhanced by ethylene treatment and *Pseudomonas* infection [40]. In a computational study [106] *Arabidopsis* PP2-A1 was shown to exhibit specificity for a diverse range of glycans including chitin oligomers and the Man_3_GlcNAc_2_ core of high mannose *N*-glycans [106,107]. Consistent with a role in plant defense, PP2-A1 represses feeding of the aphid *Myzus persicae* and aphid borne yellow virus transmission [40].

The ricin-B family of lectins contains the first lectin discovered in plants, in particular ricin. Ricin, isolated from the seeds of castor bean (*Ricinus communis*), is a chimeric protein composed of a lectin domain and a domain with RNA *N*-glycosidase activity, enabling the protein to cleave an adenine residue from the large rRNA. This enzymatic activity results in the inactivation of ribosomes and the arrest of protein translation. As a result ricin is classified in the group of ribosome inactivating proteins. The uptake of these proteins by the host cell is mediated through the ricin-B lectin domain which binds to specific glycan structures on the cell surface. The ricin-B related proteins accumulate in the vacuole or are secreted to the extracellular space [3]. Similar to the jacalin-related proteins, proteins containing a ricin-B domain are widespread and have also been identified outside the plant kingdom in animals, fungi and bacteria. These lectins are classified as R-type lectins [108]. Although the carbohydrate specificity of the ricin-B lectins can vary [109], evidence supports that many of the ricin-B related lectins play a role in defense against pathogens and insects [40]. 

## 5. Adaptive Immunity and Epigenetic Imprinting

### 5.1. Adaptive Immunity 

In vertebrates an adaptive immune response is induced following the innate immune response. When naive T-cells interact with their specific antigen presented on the surface of antigen-presenting cells, they become activated and differentiate into effector T-cells. A subset of these effector T-cells, the T-helper cells, will subsequently stimulate the proliferation and differentiation of B-cells. 

The adaptive immune response depends on the innate immune response. It was observed that several PRRs, involved in the innate immunity, also play a role in triggering the adaptive response. Toll-like receptors, nucleotide-oligomerization domain (Nod)-like receptors, retinoic acid-inducible gene-1 (RIG-1)-like receptors and some C-type lectin receptors can trigger both the innate and adaptive immune responses [110]. Whether these receptors contribute to the innate or the adaptive response is dependent of the cell type where they are expressed [111]. Moreover, lectins can have similar functions in the adaptive response and in the innate response. They play a role in cell-cell interactions and can contribute to the activation and differentiation of cells (e.g., Siglecs).

### 5.2. Epigenetic Imprinting 

Plants, unlike animals, lack the adaptive immune mechanisms and rely solely on their multilayered innate immune system to prevent pathogen infection. However, plants are not completely defenseless against reoccurring stresses.

When higher animals are exposed to a specific pathogen, the adaptive immune system creates an immunological memory after the initial response. This leads to an enhanced response to subsequent encounters with that pathogen. Although there is no adaptive immune system in plants, plants must respond and adapt to recurring biotic and abiotic stress factors especially since their sessile lifestyle does not allow to escape from these stresses. It is a well-known phenomenon that stress tolerance in plants can be improved through pre-exposure to an abiotic or biotic stress stimulus. Primed (against biotic stress) or hardened (against abiotic stress) plants display a faster and/or stronger activation to the various defense responses that are induced following infection by pathogens, attack by insects or in response to abiotic stress [112]. This priming can be elicited by either exposure to the stress clues themselves or by the exogenous application of chemical treatments [113]. The latter process can be compared to vaccination in vertebrates. The advantage of priming is that it offers the plant an enhanced protection without the costs of constitutively expressing their defense genes. However, the molecular mechanism of this stress-imprinting on plant immunity is not fully elucidated. Though several mechanisms have been proposed, Conrath *et al.* [112] suggested that two potential mechanisms can be involved. The first one involves the accumulation of signaling proteins in an inactive configuration that are activated upon stress, potentially through MAPK signaling [112,114]. The second mechanism involves the accumulation of transcription factors that enhance defense gene transcription after stress recognition [112]. Jung *et al.* [115] proposed a system in which the accumulation of secondary metabolites such as SA and azelaic acid was involved. Another mechanism was described by Bruce *et al.* [113] in which priming occurs through epigenetic changes. Epigenetics refers to the heritable changes in gene expression that do not involve changes to the DNA sequence. These epigenetic changes consist of DNA methylations, histone modifications and RNA interference that lead to changes in gene expression by activation or silencing. The links and interactions between the different mechanisms for priming are largely unknown but due to the long-lasting stress resistance, it is most likely that epigenetic mechanisms are largely in control [116].

Epigenetic changes in response to cold stress have been reported during vernalisation of *Arabidopsis*. The long-term exposure to low temperatures in winter is memorized and needed for the plant to be able to flower in the following spring [117]. This ‘memory’ involves a change in the chromatin structure of the flowering locus through modification of histones by several vernalisation (VRN) genes. Recent data suggest that also some of the stress inducible plant lectins play a role in epigenetic priming during stress response. One of the proteins involved in vernalisation in wheat is the VER2 cold inducible jacalin-related protein. Interaction with the O-GlcNAc modified TaGRP2 RNA-binding protein allows accumulation of the VRN1 transcription factor through stabilization of its mRNA [118]. Treatment of tobacco with JA, a plant hormone involved in signaling of biotic and abiotic stress and in plant development, leads to the enhanced expression of the Nictaba protein in the nucleus and the cytosol [119,120]. In addition, this lectin is also induced upon insect herbivory [120]. The accumulation of Nictaba is not limited to the treated leaf but a systemic response is observed in all leaves of the plant [121]. Screening for interaction partners revealed that the core histones H2A, H2B and H4 are the major binding partners for Nictaba in the nucleus [122]. This interaction is established through the specific binding of the lectin with O-GlcNAc modified histone proteins [123]. It was hypothesized that the interaction between Nictaba and histones bound to DNA might result in chromatin remodeling, and subsequent alteration of gene expression as a response to stress or changing environmental conditions [124].

## 6. Conclusions 

Although plants and animals shared their last common ancestor until at least one billion years ago, throughout the eons they have been facing common threats such as bacteria, fungi and viruses trying to invade them. To protect themselves against these threats, both plants and animals evolved a defense system, the immune system. While for a long time it was presumed that these defense systems in plants and animals were very different, it has become clear that both plants and animals respond to infection by pathogens using similar regulatory modules. Common features include receptors that detect molecular signatures of infectious organisms (PAMPs/MAMPs) and of tissue damage (DAMP), conserved signal transduction pathways (e.g., MAPK pathway) and the production of antimicrobial molecules. 

Lectins or carbohydrate-binding proteins play an important role in the immune system of animals and plants as PRRs. Carbohydrate structures on the surface of pathogens or released from host cells due to damage provoked by the pathogen are recognized by soluble or membrane-bound lectins that trigger a signaling cascade resulting in the induction of the defense mechanisms, the pathogen/microbial triggered immunity (P/MTI). Although in animals several lectins were found to exert a function as PRR, in plants only the LysM-domain lectins have been shown unambiguously to be dependent on carbohydrate binding for their PRR function. Other identified LecRKs or lectin receptor proteins may depend on protein-protein interactions for the recognition of their target.

In addition to the lectins with a role as PRR, lectins can play a role in stress signaling or as an effector or mediator in the stress response. In plants, several lectin families have been described that are transcriptionally upregulated upon biotic or abiotic stresses. These stress-inducible lectins mainly reside in the cytoplasm and the nucleus, and are suggested to play a role in signal transduction in several stress response pathways. 

Successful pathogens manage to overcome the innate immune response through secretion of toxins or effectors into the host cell. Plants have therefore developed another defense layer to detect these effectors via different immune receptors (resistance or R proteins) and initiate effector triggered immunity (ETI). Another method for successful pathogens to overcome the immune response is to escape degradation by the host cell. Recognition of pathogens by PRRs often leads to the phagocytosis of these pathogens by the host cell. These phagocytic vesicles are subsequently fused with lysosomes, resulting in the degradation of the pathogen. This degradation is required in vertebrate systems to activate the adaptive immunity by antigen presenting cells. However, some pathogens are capable of escaping this degradation by the delivery of effector proteins that prevent maturation of the phagocytic vacuoles. These pathogens create a niche inside these (damaged) vesicles or manage to escape these vesicles and proliferate intracellularly. Plant and animal cells have developed a mechanism to defend themselves against these intracellular parasites through autophagy. Lectins are shown to be involved in the recognition of damaged phagocytic vacuoles in animals. Since in plants the essential machineries of autophagy are conserved, lectins are suggested to play a role in this process, but at present their precise function is not fully elucidated.

Although plants lack an adaptive immune system, they are able to create long lasting stress resilience against reoccurring stresses. Epigenetic changes can facilitate quicker and more potent responses to subsequent attacks. One of the stress inducible plant lectins from tobacco, referred to as Nictaba, was shown to interact with O-GlcNAc modified histones and can potentially act as a chromatin remodeler allowing an alteration of gene expression as a result of biotic or abiotic stresses.

Although plants and animals differ significantly, their defense system relies for an important part on comparable mechanisms. This is supported by recent data which demonstrated that lectin domains that descended from common ancestors through divergent evolution appear widely across the kingdoms of life (including prokaryotes, fungi, plants and animals) [13]. However these conserved lectin domains show promiscuity in their sugar binding specificity. These variations might be linked to a directed evolution to recognize carbohydrate structures on the pathogenic agents threatening the plant/animal cell.

Despite our growing knowledge and insight into the world of lectins, the number of fully characterized lectins for which the physiological relevance has been resolved is still rather low. The presence of lectin domains as part of chimeric proteins increases the structural diversity among lectins but will also expand the functional variation within a lectin family. Future research will have to elucidate the importance of protein carbohydrate recognition for different biological processes and the key role of lectin domains in these events.
